# *In vivo* target exploration of apidaecin based on Acquired Resistance induced by Gene Overexpression (ARGO assay)

**DOI:** 10.1038/s41598-017-12039-6

**Published:** 2017-09-22

**Authors:** Ken’ichiro Matsumoto, Kurato Yamazaki, Shun Kawakami, Daichi Miyoshi, Toshihiko Ooi, Shigeki Hashimoto, Seiichi Taguchi

**Affiliations:** 1 Division of Applied Chemistry, Graduate School of Engineering, Hokkaido University, N13-W8, Kita-ku, Sapporo, 060-8628 Japan; 2 Faculty of Industrial Science and Technology, Tokyo University of Science, 102-1 Tomino, Oshamanbe-cho, Yamakoshi-gun 049-3514 Japan; 3 Present Address: Department of Chemistry for Life Sciences and Agriculture, Faculty of Life Sciences, Tokyo University of Agriculture, 1-1-1 Sakuragaoka, Setagaya-ku, Tokyo, 156-8502 Japan

## Abstract

Identifying the target molecules of antimicrobial agents is essential for assessing their mode of action. Here, we propose Acquired Resistance induced by Gene Overexpression (ARGO) as a novel *in vivo* approach for exploring target proteins of antimicrobial agents. The principle of the method is based on the fact that overexpression of the expected target protein leads to reduced sensitivity to the antimicrobial agent. We applied this approach to identify target proteins of the antimicrobial peptide apidaecin, which is specifically effective against Gram-negative bacteria. To this end, a set of overexpression *Escherichia coli* clones was tested, and peptide chain release factor 1, which directs the termination of translation, was found as a candidate, suggesting that apidaecin inhibits the termination step of translation. This finding was confirmed *in vivo* and *in vitro* by evaluating the inhibitory activity of apidaecin towards *lacZ* reporter gene expression, which is tightly dependent on its stop codon. The results of this study demonstrate that apidaecin exerts its antimicrobial effects partly by inhibiting release factors.

## Introduction

Antimicrobial peptides (AMPs) are major components of the innate immune system, and are found in many species of animals, insects, plants, and microorganisms^1^. They are expected to be useful as antimicrobial agents, because they are typically non-toxic towards eukaryotic cells and are effective against broad range of microbes including multidrug-resistant bacteria^2^.

Apidaecin is a proline-rich AMP, which was isolated from the hemolymph of honeybees immunized with bacteria and exerts static antimicrobial effects against Gram-negative bacteria^3^. It enters bacterial cells through a nonpore-forming mechanism with stereospecificity^4,5^. Although the antimicrobial activity of apidaecin is relatively low compared to that of conventional antibiotics, several studies have reported the engineering of apidaecin for enhanced antimicrobial activity^6–8^. Attempts to identify its intracellular targets have been made using *Escherichia coli* as a test strain. An assay of radioactive amino acid uptake by *E. coli* suggested that the antimicrobial action of apidaecin is to inhibit protein synthesis^9^. In addition, an intracellular target molecule of pyrrhocoricin, a homolog of apidaecin, was isolated in cell extracts using affinity-based methods. Specifically, using biotin-labeled pyrrhocoricin as a probe, one study found that the heat-shock protein DnaK is a candidate target protein^10^. In addition, pyrrhocoricin was shown to inhibit the ATPase activity of DnaK^11^. However, deletion of the *dnaK* gene in *E. coli* was not lethal. Apidaecin was also effective against Δ*dnaK* strain of *E. coli* ^12^, indicating that there might be multiple targets for apidaecin other than DnaK.

One problem with using affinity-based methods *in vitro* for determining the target molecules of AMPs is that the identified candidates may not be the true molecular targets in living cells. Another issue is that AMPs likely influence essential components of the cell, which may result in the inability to obtain the null mutant of the target molecule(s). To address these problems, we developed an *in vivo* systematic assay method using a gene overexpression library in *E. coli* to identify the target molecule(s) of antimicrobial agents. The principle of this assay method, referred to as Acquired Resistance induced by Gene Overexpression (ARGO), is based on the fact that overexpression of the target protein, if the target is protein, leads to reduced sensitivity to antimicrobial agents. Given the fact that candidates are selected from viable cells under physiological conditions, it is expected that real cellular targets will be identified with this method.

In this study, we used ARGO assay to identify the target proteins of apidaecin and to determine its mechanism of action.

## Results and Discussion

### *In vivo* assay of *E. coli* treated with apidaecin demonstrates inhibition of translation

**Figure 1:**
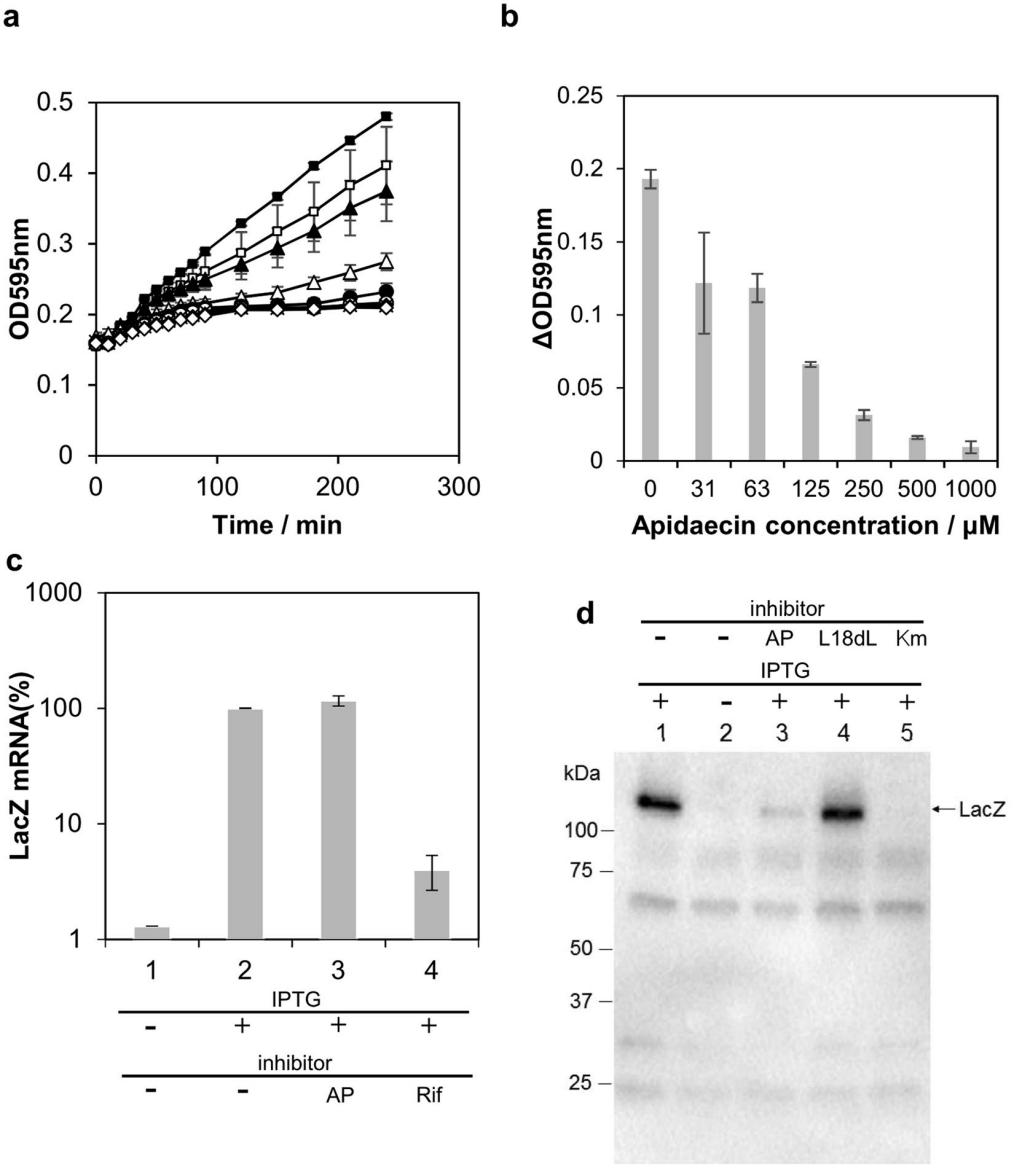
Apidaecin treatment of *E. coli* and its effects on *lacZ* expression. (**a**) Growth curve with different concentrations of apidaecin (black square: 0 μM, white square: 7.5 μM, black triangle: 15 μM, white triangle: 30 μM, black circle: 63 μM, white circle: 125 μM, cross: 250 μM, black diamond: 500 μM, white diamond: 1000 μM), (**b**) Assay of *lacZ* expression with increasing concentrations of apidaecin. *E. coli* was treated with apidaecin for 1 h, after which IPTG was added to induce *lacZ* expression. ΔOD_595_ was defined as the difference between optical density (OD) with and without X-gal. (**c**) Quantification of *lacZ* mRNA expression by quantitative real-time PCR. 1: untreated, 2: addition of 0.1 mM IPTG, 3: treatment with 500 μM apidaecin and 0.1 mM IPTG, and 4: treatment with 25 μM rifampicin and 0.1 mM IPTG. The expression level was defined as relative to the value in treatment No. 2. (**d**) Immunoblot analysis of native LacZ. Lane 1: addition of 0.1 mM IPTG, lane 2: untreated, lane 3: 500 μM apidaecin and 0.1 mM IPTG, lane 4: 500 μM L18dL apidaecin and 0.1 mM IPTG, and lane 5: 100 μM kanamycin and 0.1 mM IPTG. Data are expressed as the mean ± standard deviation of three trials (A, B, and C).

To determine which process is inhibited by apidaecin (i.e., transcription or translation), the effects of apidaecin on protein synthesis were monitored using *lacZ* as a reporter gene. *E. coli* were treated with different concentrations of apidaecin for 1 h, after which isopropyl β-D-1-thiogalactopyranoside (IPTG), an inducer of *lacZ* expression, was added. Dose-dependent inhibition of β-galactosidase (encoded by *lacZ*)-mediated chromogenesis was observed, and the addition of 500 μM apidaecin almost completely inhibited β-galactosidase-mediated chromogenesis as well as cell growth (Fig. 1a,b). Rifampicin and kanamycin, well-known inhibitors of transcription and translation, respectively, were used as positive controls (Fig. S1). The effects of apidaecin on transcription were determined by quantification of *lacZ* mRNA levels. After confirming the IPTG-induced increase in *lacZ* mRNA synthesis (Fig. 1c, No. 2), no significant influence was observed on mRNA transcription with 500 μM apidaecin (Fig. 1c, No. 3). In contrast, β-galactosidase protein synthesis under the same condition was considerably diminished, as determined by immunoblot analysis (Fig. 1d), indicating that inhibition of β-galactosidase-mediated chromogenesis was caused by diminished protein synthesis and not by the inhibition of enzymatic activity and/or folding of the LacZ peptide into a mature protein. L18dL apidaecin 1b, which contains the D-Leu residue at position 18 in highly conserved C-terminal region, was designed based on L17D-L mutant of apidaecin Ho + (GKPRPQQVPPRPPHPRL), the antibacterial activity toward *E. coli* of which was 0.1-fold compared to wild type peptide^9^. Addition of 500 μM L18dL exhibited no antimicrobial effect against *E. coli* (data not shown), and thus, was used as the negative control. These results demonstrate that apidaecin inhibits translation, which is in accordance with several recent reports, which showed that proline-rich peptides such as apidaecin^9^, oncocin^13^, bactenecin, 7^14^ and pyrrhocoricin^15^ inhibit translation.

### *In vivo* systematic assay of apidaecin target molecule(s) based on ARGO

**Figure 2:**
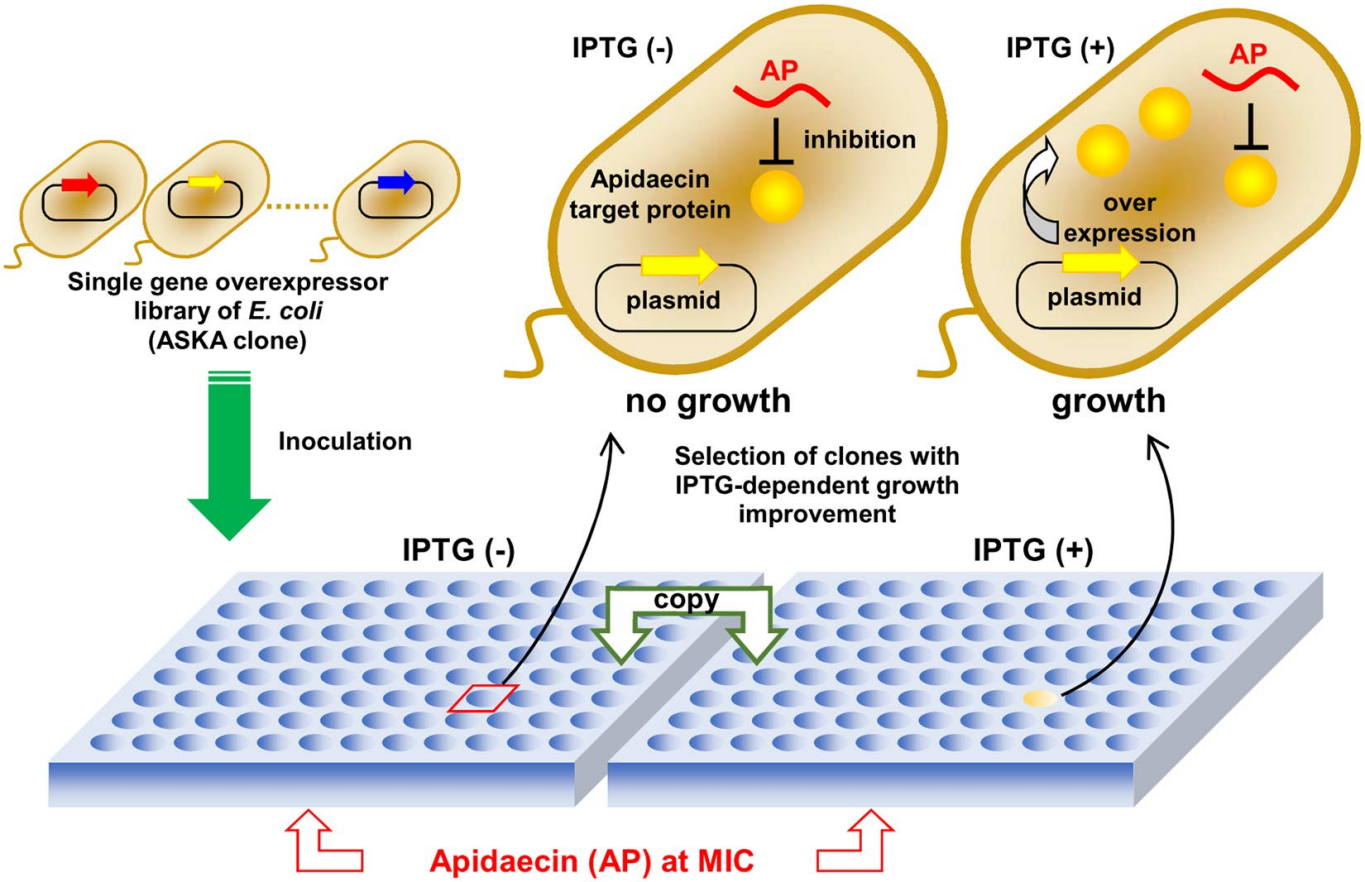
*In vivo* assay of apidaecin target proteins using the ARGO method in *E. coli*. The ASKA clone is a complete set of open reading frame clones of *E. coli*. Cells were cultivated in the presence of apidaecin at the minimal inhibitory concentration. Each plasmid-encoded candidate gene was expressed under control of IPTG-inducible lac promoter. The overexpressors, the gene products of which are directly or indirectly involved in the action of apidaecin, may exhibit reduced sensitivity against apidaecin depending on IPTG induction. ARGO assay was used to evaluate each overexpressor for sensitivity against apidaecin. The genes involved in translation were tested.

To identify protein(s) involved in apidaecin-mediated inhibition of translation, we designed the *in vivo* ARGO assay method (Fig. 2), based on the principle that recombinant proteins overexpressed in *E. coli* (A complete Set of *E. coli* K-12 open reading frame Archive [ASKA])^16^ will exhibit resistance against their target antimicrobial agents, thereby allowing identification of proteins involved in the action of antimicrobial agents of interest. As positive controls, we used two antimicrobial agents, triclosan for which the target is enoyl-ACP reductase (FabI), which is involved in fatty acid synthesis^17^, and trimethoprim for which the target is dihydrofolate reductase (FolA), which is involved in folic acid synthesis. As expected, overexpression of the FabI and FolA target proteins reduced the sensitivity of *E. coli* to their corresponding antimicrobial agents (Fig. S2), indicating that ARGO may be useful for identifying apidaecin targets.

**Figure 3:**
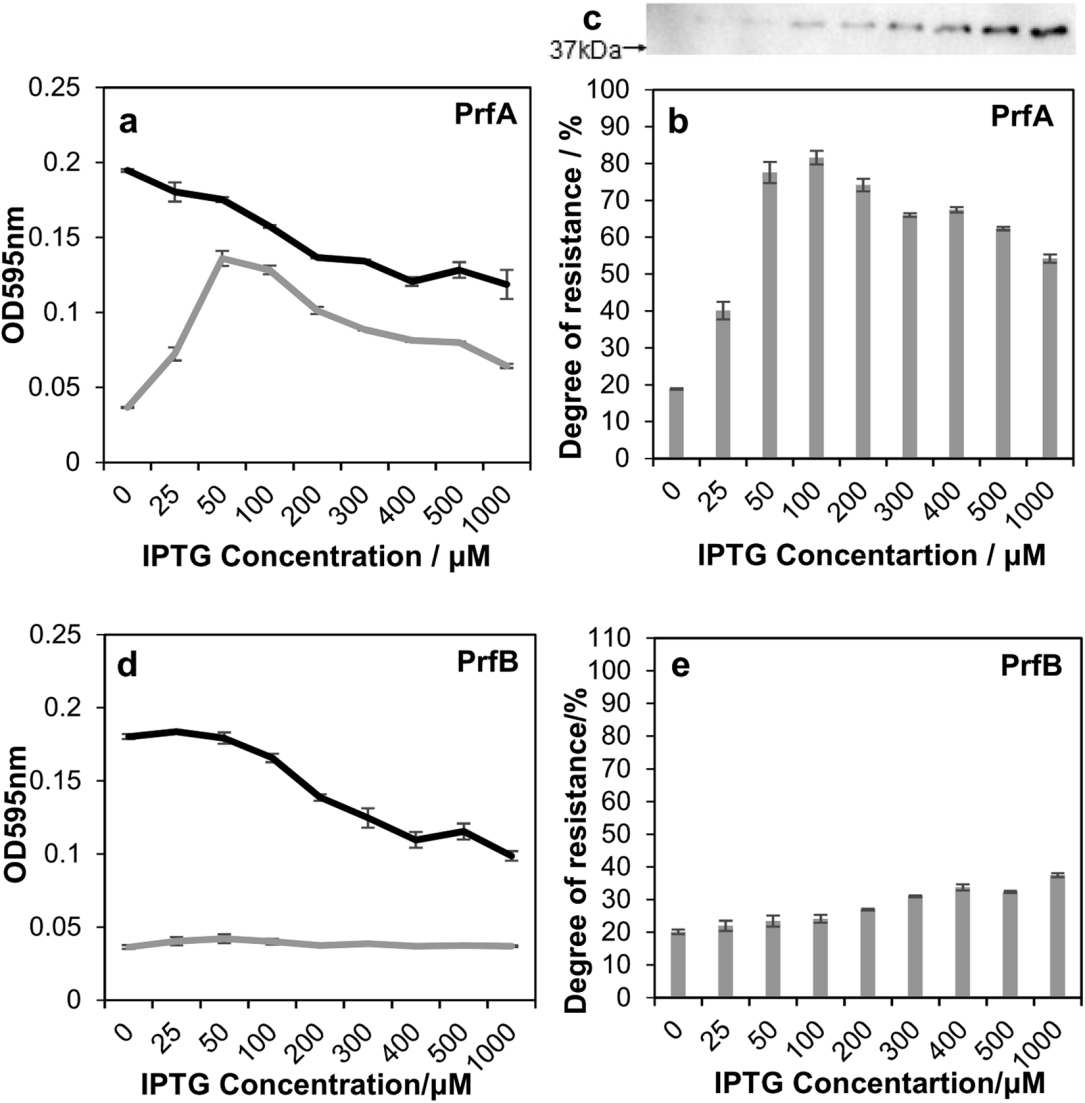
Resistance of Release factors-overexpressing *E. coli* against apidaecin, as determined by ARGO assay. (**a**) Cell growth of recombinant *E. coli* harboring the *prfA* gene with increasing IPTG concentration. Black line: no apidaecin added. Gray line: 6.25 μM apidaecin added. (**b**) The degree of resistance against apidaecin upon PrfA overexpression. (**c**) Immunoblot analysis of exogenous PrfA detected using the anti-His-tag antibody. Native PrfA was not detected by analysis, so no band was detected without IPTG addition. (**d**) Cell growth of recombinant *E. coli* harboring the *prfB* gene with increasing IPTG concentration. (**e**) The degree of resistance against apidaecin upon PrfB overexpression. Data are expressed as the mean ± standard deviation of three trials.

We performed ARGO assay for a set of recombinant *E. coli* overexpressing 64 essential proteins involved in translation, using data from the COG database (http://www.ncbi.nlm.nih.gov/COG/). Only one protein, peptide chain release factor 1 (PrfA)^18^, was selected as a positive candidate; its overexpression resulted in *E. coli* resistance against apidaecin of up to 80% (Fig. 3b). The overexpression of PrfA alone retarded cell growth. Therefore, the degree of resistance was determined based on the relative cell growth with and without apidaecin at the same concentration of IPTG. The addition of >200 μM IPTG did not further increase resistance that occurred upon PrfA overexpression (Fig. 3a). Overexpression of PrfB (Fig. 3d) and other translation-related proteins tested in the assay (Table S1) did not promote cell growth in the presence of apidaecin.

### *In vivo* inhibitory activity of apidaecin for protein expression is stop codon-dependent

**Figure 4:**
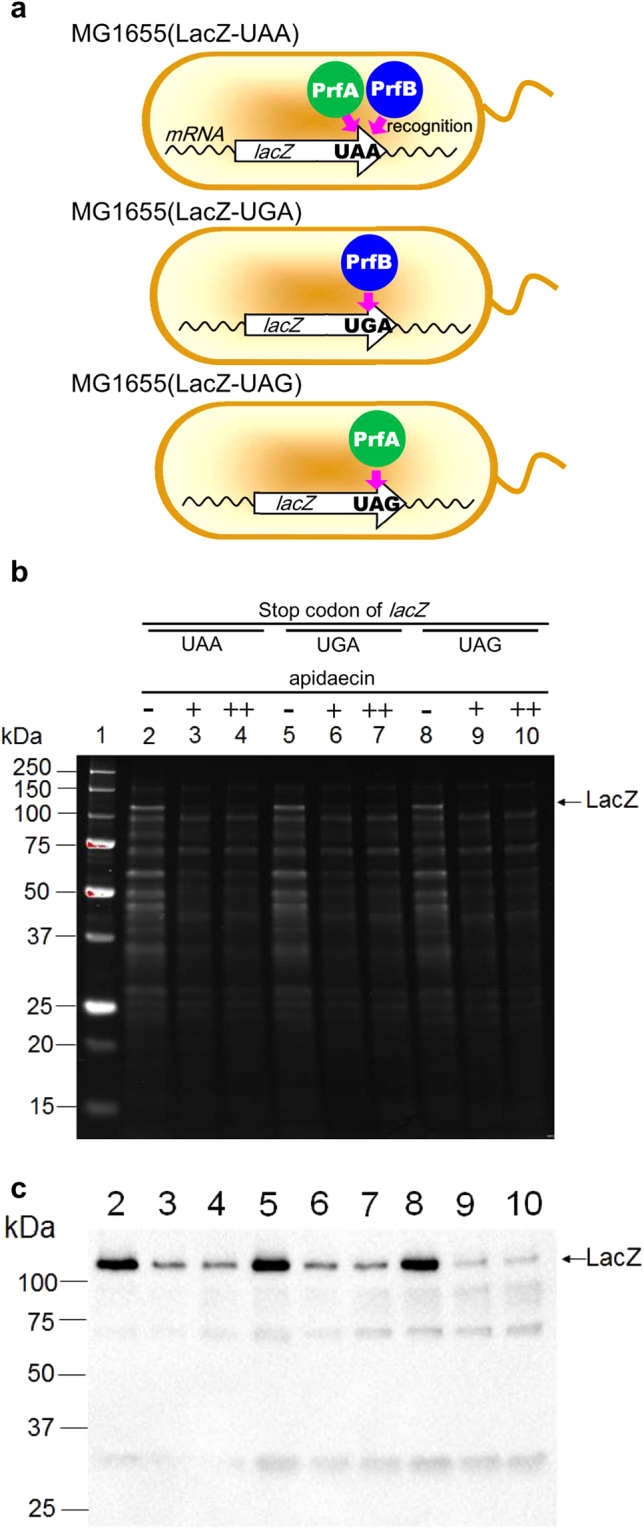
*In vivo* inhibition of lacZ expression with different stop codons. (**a**) The strains used in this assay. Genome-encoded *lacZ* gene was modified to possess three stop codons, which were recognized by PrfA and/or PrfB. (**b**) SDS-PAGE and (**c**) immunoblot using anti-LacZ. Lane 1: Size marker, lanes 2, 3 and 4: MG1655(*lacZ*-UAA), 5, 6 and 7: MG1655(*lacZ*-UGA), and 8, 9 and 10: MG1655(*lacZ*-UAG). Lanes 3, 6, and 9: 250 μM apidaecin was added. Lanes 4, 7 and 10: 500 μM apidaecin was added. The amount of protein was normalized to the OD of the cells.

The selection of PrfA as a putative apidaecin target suggested that apidaecin may inhibit the termination step of translation. Accordingly, it was predicted that the stop codon influences the inhibitory efficiency of apidaecin. To test this hypothesis, three *E. coli* strains, MG1655(LacZ-UAA), MG1655(LacZ-UGA), and MG1655(LacZ-UAG), which possess the *lacZ* gene with different stop codons, were subjected to an apidaecin inhibitory assay. The UAA stop codon was recognized by both release factors, but UGA was only recognized by PrfB and UAG was only recognized by PrfA^18^. For all of the constructs, treatment with apidaecin decreased expression levels of whole proteins (Fig. 4a), consistent with the observed inhibitory effects of apidaecin on protein synthesis. Notably, immunoblot analysis demonstrated that the degree of inhibition of *lacZ* expression by apidaecin was dependent on the stop codon; indeed, the UAG stop codon led to considerably stronger inhibition than the other constructs (Fig. 4b and reproduction data in Fig. S3). These results indicate that apidaecin is involved in the inhibition of termination step of translation, supporting our hypothesis that PrfA is a target of apidaecin. PrfB-mediated termination was less efficiently inhibited by the action of apidaecin. In addition, it should be noted that the addition of apidaecin reduced the intensity of the LacZ band, but the observed molecular weight of the protein remained unchanged. This indicates that protein expression was inhibited at the termination and/or initiation steps rather than during elongation of the peptide.

### *In vitro* assay of the action of apidaecin using the PURE system

**Figure 5:**
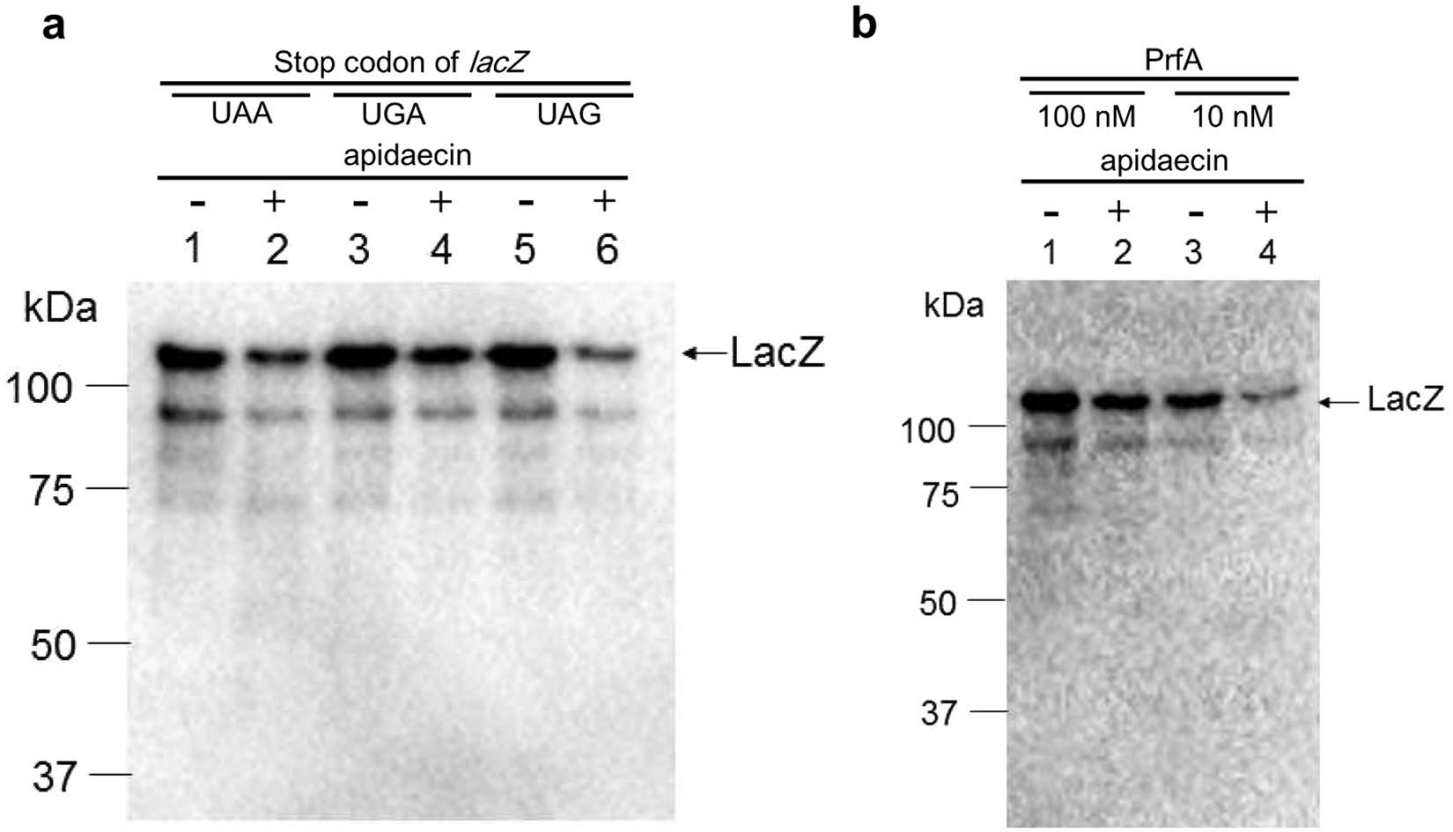
(**a**) *In vitro* inhibitory assay of lacZ expression with different stop codons. Lanes 1 and 2; *lacZ*-UAA, lanes 3 and 4; *lacZ*-UGA, and lanes 5 and 6; *lacZ*-UAG. Lanes 2, 4, and 6; 200 μM apidaecin added. (**b**) *In vitro* inhibitory assay of *lacZ* (UAG) expression by apidaecin with modified concentrations of release factors. PrfB was omitted in all conditions. Lanes 1 and 2; 100 nM PrfA, lanes 3 and 4; 20 nM PrfA. Lanes 1 and 3; no apidaecin, lanes 2 and 4; 200 μM apidaecin.

*In vivo* experiments indicated that inhibition of protein synthesis by apidaecin was stop codon-dependent. To determine whether this inhibition was due to the direct interaction of apidaecin with the translation machinery, the *in vitro* transcription-translation system (PURE system) was used for elucidating the effects of the stop codons on the inhibitory efficiency of apidaecin. Three template DNA encoding *lacZ* with three stop codons were used for the analysis. The results showed that apidaecin exhibited inhibitory effects in the PURE system (Fig. 5a) as well i*n vivo*. More importantly, the inhibition efficiencies were different depending upon the stop codon expressed. The UAG stop codon led to the most efficient inhibition by apidaecin, which was consistent with the *in vivo* data. These results support our hypothesis that apidaecin inhibits the termination step of translation. To further evaluate the relationship between PrfA and apidaecin, the inhibition of *in vitro* protein synthesis was examined with a reduced concentration of PrfA. The inhibition ratio of *lacZ* (UAG) expression (45%) at the low PrfA concentration was greater than that (21%) observed at the standard PrfA concentration (Fig. 5b). Therefore, the efficiency of translation inhibition by apidaecin was dependent upon the concentration of PrfA. The results of this *in vitro* inhibitory assay were consistent with those obtained from *in vivo* assay.

### Proposed model for mechanism of action of apidaecin

**Figure 6:**
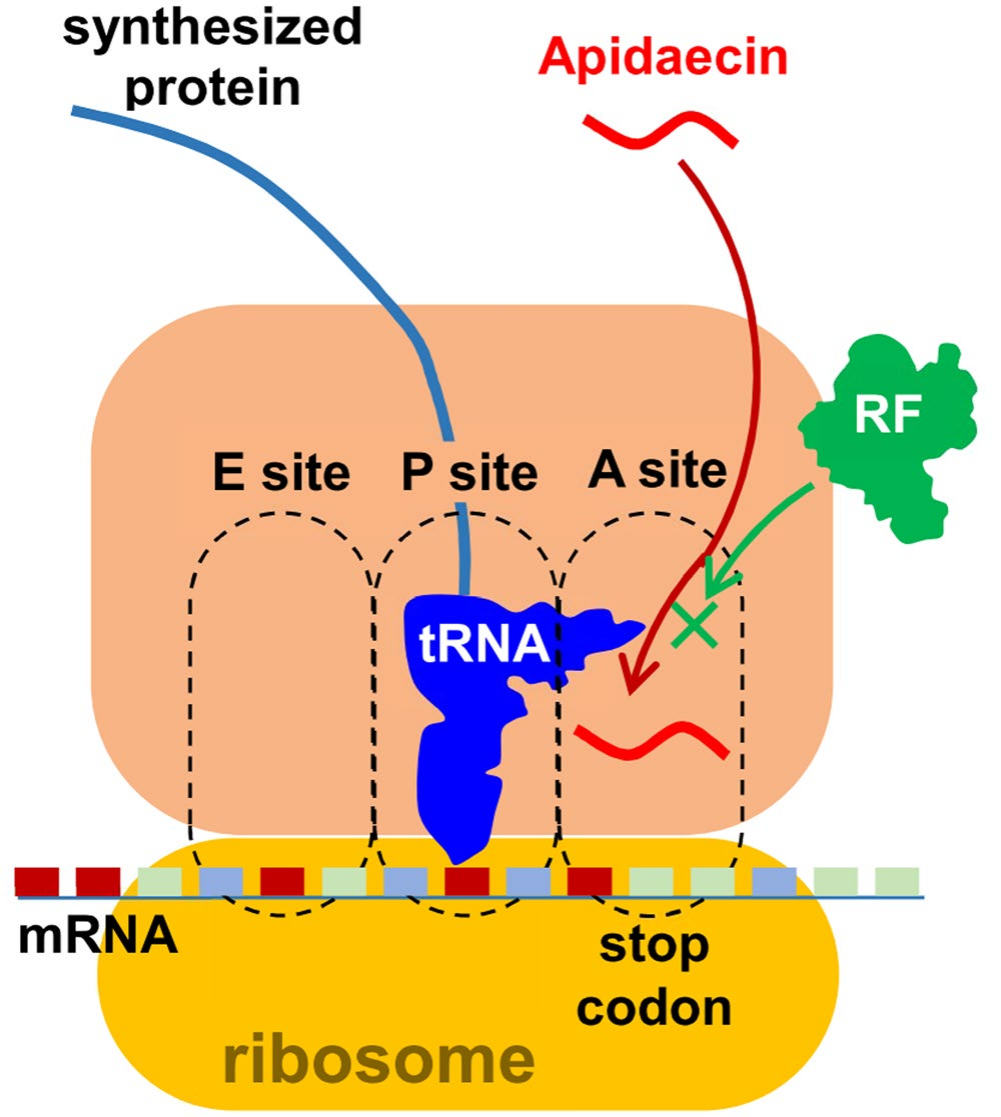
Proposed model of target inhibition by apidaecin. Apidaecin competitevly binds with release factors (RF) to the A-site of ribosomes, and inhibits the termination step of translation. This accounts for the stop codon-dependent inhibition of translation by apidaecin *in vivo* (Fig. 4c) and *in vitro* (Fig. 5a). Overexpression of PrfA (RF1) reduced sensitivity against apidaecin (Fig. 3b), presumably because the binding of apidaecin to ribosome is reversible. This could be the reason for the static antimicrobial effects of apidaecin.

PrfA binds to the A-site on ribosomes and recognizes stop codons in mRNA for the termination of translation. Therefore, a putative mechanism underlying the action of apidaecin is that it binds to the A-site of ribosomes competitively with PrfA. In fact, a recent crystallography study demonstrated that the proline-rich antimicrobial peptide bactenecin 7 binds to the A-site of ribosomes^14^. Moreover, apidaecin and other proline-rich peptides have also been shown to bind to ribosomes^19^. Based on these reports, we propose a model of the potential action of apidaecin in Fig. 6, namely, that apidaecin may exert its antimicrobial effects, at least in part, by competing for binding to the A-site with PrfA (Fig. 6). To the best of our knowledge, this is the first report to demonstrate that AMPs inhibit the termination step of translation. In addition, this unique property makes it possible for apidaecin to synergize with other antibiotics that have different modes of action, for enhanced antimicrobial effects.

The assay method described in this study permits the identification of target proteins of apidaecin based on sensitivity to the peptide. Previous attempts to identify apidaecin target molecule(s) were made based on the postulation that apidaecin will exhibit affinity with its target molecule(s) *in vitro* ^10,20^. This affinity-based approach did not identify PrfA as a target, because PrfA does not necessarily possess direct binding affinity to apidaecin based on our proposed mechanism. Another strategy has been to generate natural resistance of *E. coli* against apidaecin^21^, which also did not identify PrfA as a candidate, probably because the overexpression of PrfA retards the cell growth.

## Conclusions

In this study, *in vivo* systematic assay using the ARGO method was developed and applied to the identification of apidaecin targets. PrfA was identified as a novel target molecule of apidaecin. Indeed, apidaecin inhibited gene translation with expression of the UAG stop codon, which was only recognized and terminated by PrfA. Based on these findings, we propose a new mechanism of antimicrobial activity, in which apidaecin inhibits the termination step of ribosomal translation by blocking release factors. The ARGO assay should be applicable to a broad range of antimicrobial agents to reveal their target proteins and their mechanisms of actions.

## Methods

### Peptide preparation

Apidaecin Ib GNNRPVYIPQPRPPHPRL and related peptides were purchased from Bex Corp. (Tokyo, Japan) and purified using preparative high-performance liquid chromatography (JASCO engineering, Hachioji, Japan) equipped with a TSKgel ODS-80Ts column (TOSOH, Tokyo, Japan). The fractionated peptides were subjected to matrix assisted laser desorption ionization-time of flight mass spectrometry (Microflex, Bruker Daltonics, Billerica, MA, USA) using α-cyano-4-hydroxycinnamic acid as a matrix to confirm the molecular weight. The concentrations of the peptides were determined using an amino acid analyzer (L-8900, Hitachi, Tokyo, Japan) and post-labeling method with ninhydrin^22^.

### *E*. *coli* cultivation conditions

#### Procedure A

*E*. *coli* were grown on 2 mL LB medium in a test tube containing 10 g/L trypton, 5 g/L Bacto yeast extract, and 10 g/L sodium chloride, with reciprocal shaking at 175 rpm at 30 °C for 14 h. A total of 20 μL culture medium was transferred into 2 mL fresh LB medium and incubated at 30 °C until the OD at 600 nm reached 0.2.

#### Procedure B

The cells from Procedure A were washed with 2 mL PB medium containing 10 g/L trypton and 9 g/L sodium chloride, and re-suspended in 1 mL PB medium. A total of 80 μL medium was combined with 20 μL antimicrobial agents, followed by incubation in a 96-well plate at 30 °C for 4 h with shaking at 1200 rpm.

#### Procedure C

The 96-well plate culture was started following Procedure B. After 60 min, 0.1 mM IPTG was added and the plate was incubated for an additional 60 min.

#### Procedure D

The 96-well plate culture was started as well as Procedure B with a modification that the cells were resuspended in 1 mL PB medium containing 100 μg/ml 5-bromo-4-chloro-3-indolyl-β-D-galactopyranoside (X-gal). After 60 min, 0.1 mM IPTG was added and the plate was further incubated for 120 min. The OD of the culture in the test tube was measured using a UV-VIS spectrophotometer (Genesys20, Thermo Scientific, Waltham, MA, USA) at 600 nm, and OD in the 96-well plate was monitored at 595 nm using an iMark Microplate Absorbance Reader (Biorad, Hercules, CA, USA).

### Inhibition assay of LacZ expression

*E. coli* MG1655 cells were cultivated using Procedure D. The activity of LacZ was estimated based on the subtraction of ODs at 595 nm obtained with and without supplement of X-gal.

### Real-time reverse transcriptase-PCR

The *E. coli* strain MG1655 from Procedure C were harvested by centrifugation at 4 °C, and total RNA was extracted using Isogen (Nippongene, Toyama, Japan) according to the manufacturer’s instructions. The reverse transcriptase reaction was performed using PrimeScript RT-PCR Kit (Takara, Tokyo, Japan) based on the provided protocol. The *lacZ* fragment was amplified using a pair of primers: lacZ_RTf1, ACTGACGGGCTCCAGGAGTCGT and lacZ_RTr1, with AGTTCAACATCAGCCGCTACAGTCA used as the standard. The standard curve was made in the range of 10^−3^ to 10^−7^ ng DNA/μL. A total of 6 μL reverse transcriptase reaction product was combined with 10 μL TaqMan Gene Expression Master Mix (Applied Biosystems, Foster City, CA, USA), 1 μL lacZ_RTf1, 1 μL lacZ_RTr1, and 2 μL TaqMan probe lacZ_2116, GTCGATATTCAGCCATGTGCCTTCTTCCGCGTGCAG, which was bound to fluorescein as a reporter and NFQ-MGB as a quencher. The samples were prepared in triplicate and transferred to a MicroAmp Fast Optical 48-well Reaction Plate (Applied Biosystems) and sealed with a MicroAmp Optical 48-well Adhesive Film. The plate was centrifuged at 1,970 g for 10 min at 4 °C. Real-time PCR (qPCR) was performed using the StepOne real-time PCR system (Applied Biosystems). The thermal cycle program was as follows: 50 °C for 2 min, 95 °C for 10 min, 40 cycles of 95 °C for 15 s, and 60 °C for 1 min.

### Immunoblot analysis

The *E. coli* strain MG1655 from Procedure C were harvested and suspended in lysis buffer containing 50 mM sodium phosphate (pH 7.0), 10 mM imidazol, and 300 mM NaCl. The OD was normalized using lysis buffer, after which the crude extract was prepared using sonication on ice 5 s × 5 times. Anti-β-galactosidase antibody (Biotin) ab6645 was purchased from abcam (Cambridge, UK) and used at a 10^5^-fold dilution. Immun-Star Goat Anti-Rabbit (GAR)-HRP Conjugate (Biorad) was used as the secondary antibody. Bands were detected using the ECL Select Western Blotting Detection Reagent (GE Healthcare, Little Chalfont, UK) and were recorded on ChemiDox XRS (Biorad). Band density was quantified using ImageJ software.

### *In vivo* apidaecin resistance assay using single-gene overexpressing recombinants of *E. coli* (ASKA clone)

The ASKA clone is a plasmid collection, which encompasses 4123 genes of the *E. coli* chromosome. The recombinant *E. coli* strain JM109 harboring pCA24N derivatives were grown on 2 mL LB medium containing 30 µg/mL chloramphenicol in a test tube, with reciprocal shaking at 175 rpm at 37 °C for 18 h. A total of 80 μL culture medium was transferred into fresh 2 mL LB medium containing 30 µg/mL chloramphenicol and different concentrations of IPTG (see Supplementary Information for detail), and further incubated at 37 °C with until the OD at 600 nm reached 0.4. The pre-culture was diluted 10^5^ times with PB medium containing 30 µg/mL chloramphenicol so that the initial cell density was approximately 1000 colony forming units/mL. Then a 50 µL aliquot was combined with a 50 µL mixture of apidaecin and IPTG (final concentration of apidaecin was 6.25 µM). As a control, 50 µL IPTG and 50 µL sterilized water were added, respectively. The cells were cultivated in 96-well plates using a microplate shaker (BioShaker M-BR-024 Taitec, Shizuoka, Japan) with shaking at 1200 rpm at 30 °C for 18 h. Several recombinants that exhibited severe growth inhibition upon addition of 0.1 mM IPTG, were incubated with decreased concentrations of IPTG (Table S1). Cell growth in a 96-well plate was optically measured using a microplate reader (iMark Microplate Absorbance Reader, Bio-rad) at a wavelength of 595 nm. The degree of resistance against apidaecin was defined as the OD obtained with the the addition of IPTG and apidaecin divided by the OD with the addition of IPTG only.

1 $${\rm{Degree}}\,{\rm{of}}\,{\rm{resistance}}=\frac{{\rm{OD}}\,{\rm{with}}\,{\rm{apidaecin}}\,{\rm{and}}\,{\rm{IPTG}}}{{\rm{OD}}\,{\rm{with}}\,{\rm{IPTG}}}$$

The selected cells were pre-cultured with 25 µM IPTG. Then the pre-cultures were inoculated into PB medium containing 6.25 µM apidaecin and different concentrations of IPTG in a 96-well microplate, followed by incubation at 30 °C for 18 h. Triclosan (Irgasan) and trimethoprim were dissolved in ethanol and methanol, respectively, and filtered prior to use.

### *In vivo* inhibitory assay of lacZ expression with different stop codons

The C-terminal region of the *lacZ* gene in *E. coli* chromosomal DNA was replaced with three DNA fragments containing three stop codons (Fig. S4). The DNA fragments including a flippase recognition target (FRT) cassette with a kanamycin-resistant gene, and the C-terminal sequence of the *lacZ* gene with three stop codons, were amplified from pKD13 using three forward primers: TGAGCGCCGGTCGCTACCATTACCAGTTGGTCTGGTGTCAAAAATAATGTAGGCTGGAGCTGCTTCG, TGAGCGCCGGTCGCTACCATTACCAGTTGGTCTGGTGTCAAAAATAGTGTAGGCTGGAGCTGCTTCG, and TGAGCGCCGGTCGCTACCATTACCAGTTGGTCTGGTGTCAAAAATGATGTAGGCTGGAGCTGCTTCG (stop codons are underlined), and a reverse primer: ATAATGGATTTCCTTACGCGAAATACGGGCAGACATGGCCTGCCCGGATTCCGGGGATCCGTCGACC. The template DNA in the PCR products was removed by *Dpn*I treatment. *E. coli* strain MG1655 were grown on LB medium containing 0.2% arabinose until the OD reached 0.4, after which the cells were washed with sterilized water and re-suspended in sterilized water. The cells were transformed by electroporation at 1.8 kV using pKD46^23^, which is a temperature-sensitive plasmid harboring the red recombinase gene under the control of P_Bad_ promoter. The cells selected on LB containing 0.2% arabionse and 100 µg/ml ampicilline at 30 °C were further transformed by electroporation using the PCR products. Recombinant cells, which grew on LB containing 0.2% arabinose and 50 µg/ml kanamycin at 30 °C, were incubated in 2 mL LB medium at 37 °C for 24 h to induce the loss of pKD46 plasmid. The cells exhibiting resistance to kanamycin and sensitivity to ampicillin were chosen as candidates. Then pCP20, which harbors the flippase gene, was introduced into cells to remove the kanamycin cassette between FRT sequences. The colonies, which were sensitive to kanamycin, were chosen and incubated in LB medium at 42 °C for 24 h to induce the loss of pCP20. Ampicilline-sensitive colonies were chosen and recombination in their chromosomal DNA was evaluated by colony PCR and sequencing. The obtained strains were referred as MG1655(*lacZ*-UAA), MG1655(*lacZ*-UGA), and MG1655(*lacZ*-UAG), respectively. The recombinant strains, MG1655(*lacZ*-UAA), MG1655(*lacZ*-UGA), and MG1655(*lacZ*-UAG), were cultivated via Procedure A and C, and subjected to immunoblot analysis as described above. Five wells in a 96-well plate were prepared for each genotype.

### Inhibitory assay using the *in vitro* transcription-translation system (PURE system)

The *lacZ* gene fragments, which possess three stop codons; TAA, TGA and TAG, and contain T7 promoter sequence, were amplified by PCR using pCA24N-lacZ, which was a part of ASKA clone, as a template, and following primers; forward primer 1, AAGGAGATATACCAATGACCATGATTACGGA, and reverse primers, TATTCATTATTTTTGACACCAGACC, TATTCATCATTTTTGACACCAGACC and TATTCACTATTTTTGACACCAGACC (stop codons were underlined). The PCR products were purified by agarose gel extraction and used for second PCR to add the T7 promoter sequence using a forward primer 2, GAAATTAATACGACTCACTATAGGGAGACCACAACGGTTTCCCTCTAGAAATAATTTTGTTTAAGAAGGAGATATACCA, and aforementioned reverse primers. The amplified DNA was treated with chloroform/phenol/isoamyl alcohol, concentrated by ethanol precipitation, and diluted to 100 ng/μl using RNase free water. The DNA solution was used for *in vitro* transcription-translation reaction using PUREfrex ver 1.0 (GeneFrontier Corp., Chiba, Japan). The solution I, II, and III provided by the kit protocol was combined with the apidaecin solution to contain 0 to 200 μM apidaecin. The PUREfrex reaction with modified releasing factors was performed using a custom-design kit, in which solution II does not contain T7 RNA polymerase, RF1, RF2, RF3 and RRF. Ten microliter solution I, 1 μL solution II, 1 μL solution III, 0.1 μL 6 μM T7 RNA polymerase, 0.5 μL 4 or 0.8 μM RF1, 0.5 μL 4 μM RF3, 0.5 μL 40 μM RRF, and 0–4 μL 1 mM apidaecin were combined and diluted to 19 μL with RNAse free water. The mixture was incubated at room temperature for 15 min, and 1 μL template DNA (LacZ [UAG]) was added. Then the mixture was further incubated at 37 °C from 3 h. Then 1 μL reaction mixture was used for immnoblot analysis.

### Data availability

All data generated or analysed during this study are included in this published article and its Supplementary Information files.

## Electronic supplementary material

Supplementary information

Table S1

## References

[1] Toke O (2005). Antimicrobial peptides: New candidates in the fight against bacterial infections. Biopolymers.

[2] Li Y, Xiang Q, Zhang Q, Huang Y, Su Z (2012). Overview on the recent study of antimicrobial peptides: origins, functions, relative mechanisms and application. Peptides.

[3] Casteels P, Ampe C, Jacobs F, Vaeck M, Tempst P (1989). Apidaecins - Antibacterial peptides from honeybees. Embo Journal.

[4] Casteels P, Tempst P (1994). Apidaecin-type peptide antibiotics function through a non-poreforming mechanism involving stereospecificity. Biochemical and Biophysical Research Communications.

[5] Mattiuzzo M (2007). Role of the *Escherichia coli* SbmA in the antimicrobial activity of proline-rich peptides. Mol Microbiol.

[6] Taguchi S, Mita K, Ichinohe K, Hashimoto S (2009). Targeted engineering of the antibacterial peptide apidaecin, based on an in iivo monitoring assay system. Appl. Environ. Microbiol..

[7] Matsumoto K (2010). Flow cytometric analysis of the contributing factors for antimicrobial activity enhancement of cell-penetrating type peptides: case study on engineered apidaecins. Biochem Biophys Res Commun.

[8] Bluhm ME (2016). N-terminal Ile-Orn- and Trp-Orn-motif repeats enhance membrane interaction and increase the antimicrobial activity of apidaecins against *Pseudomonas aeruginosa*. Front Cell Dev Biol.

[9] Castle M, Nazarian A, Yi SS, Tempst P (1999). Lethal effects of apidaecin on *Escherichia coli* involve sequential molecular interactions with diverse targets. J Biol Chem.

[10] Otvos L (2000). Interaction between heat shock proteins and antimicrobial peptides. Biochemistry.

[11] Kragol G (2001). The antibacterial peptide pyrrhocoricin inhibits the ATPase actions of DnaK and prevents chaperone-assisted protein folding. Biochemistry.

[12] Berthold N, Hoffmann R (2014). Cellular uptake of apidaecin 1b and related analogs in Gram-negative bacteria reveals novel antibacterial mechanism for proline-rich antimicrobial peptides. Protein Pept Lett.

[13] Krizsan A (2014). Insect-derived proline-rich antimicrobial peptides kill bacteria by inhibiting bacterial protein translation at the 70S ribosome. Angew Chem Int Ed Engl.

[14] Gagnon MG (2016). Structures of proline-rich peptides bound to the ribosome reveal a common mechanism of protein synthesis inhibition. Nucleic Acids Res..

[15] Taniguchi M (2016). Pyrrhocoricin, a proline-rich antimicrobial peptide derived from insect, inhibits the translation process in the cell-free Escherichia coli protein synthesis system. J Biosci Bioeng.

[16] Kitagawa M (2005). Complete set of ORF clones of Escherichia coli ASKA library (a complete set of E. coli K-12 ORF archive): unique resources for biological research. DNA Res.

[17] Hoang TT, Schweizer HP (1999). Characterization of *Pseudomonas aeruginosa* enoyl-acyl carrier protein reductase (FabI): a target for the antimicrobial triclosan and its role in acylated homoserine lactone synthesis. J Bacteriol.

[18] Scolnick E, Tompkins R, Caskey T, Nirenberg M (1968). Release factors differing in specificity for terminator codons. Proc. Natl. Acad. Sci. U S A.

[19] Krizsan A, Prahl C, Goldbach T, Knappe D, Hoffmann R (2015). Short proline-rich antimicrobial peptides inhibit either the bacterial 70S ribosome or the assembly of its large 50S subunit. Chembiochem.

[20] Volke D, Krizsan A, Berthold N, Knappe D, Hoffmann R (2015). Identification of Api88 binding partners in *Escherichia coli* using a photoaffinity-cross-link strategy and label-free quantification. J Proteome Res.

[21] Schmidt R, Krizsan A, Volke D, Knappe D, Hoffmann R (2016). Identification of new resistance mechanisms in *Escherichia coli* against apidaecin 1b using quantitative gel- and LC-MS-based proteomics. J Proteome Res.

[22] Spackman DH, Stein WH, Moore S (1958). Automatic recording apparatus for use in chromatography of amino acids. Anal.Chem..

[23] Datsenko KA, Wanner BL (2000). One-step inactivation of chromosomal genes in Escherichia coli K-12 using PCR products. Proc. Natl. Acad. Sci. USA.

