# Supplementary material for: In vivo target exploration of apidaecin based on Acquired Resistance induced by Gene Overexpression (ARGO assay)
Source: Sci Rep. 2017 Sep 22;7:12136. doi: 10.1038/s41598-017-12039-6 (PMC5610309; doi:10.1038/s41598-017-12039-6)
Supplement: Supplementary file 1 — Supplementary information [file 41598_2017_12039_MOESM1_ESM.pdf]

Supplemental Information

to

In vivo target exploration of apidaecin based on Acquired Resistance induced by Gene Overexpression (ARGO assay)

Ken'ichiro Matsumoto [1,*], Kurato Yamazaki [1], Shun Kawakami [1], Daichi Miyoshi [1],

Toshihiko Ooi [1], Shigeki Hashimoto [2], and Seiichi Taguchi [1,†,*]

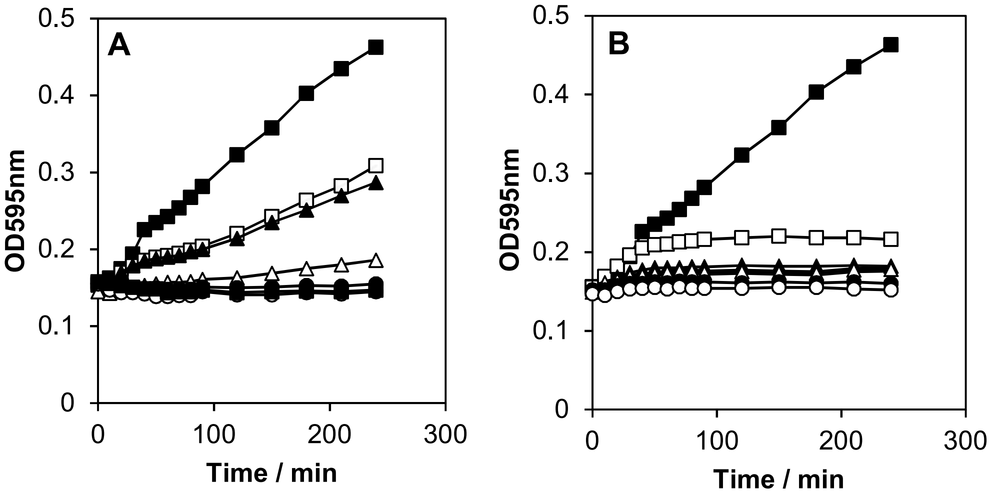

Figure S1. Growth curve with different concentrations of rifampicin (A) and kanamycin (B). (A) Black square: 0 μM, white square: 3.1 μM, black triangle: 6.3 μM, white triangle: 12.5 μM, black circle: 25 μM, white circle: 50 μM, cross: 100 μM, black diamond: 500 μM. (B) Black square: 0 μM, white square: 12.5 μM, black triangle: 25 μM, white triangle: 50 μM, black circle: 100 μM, white circle: 200 μM.

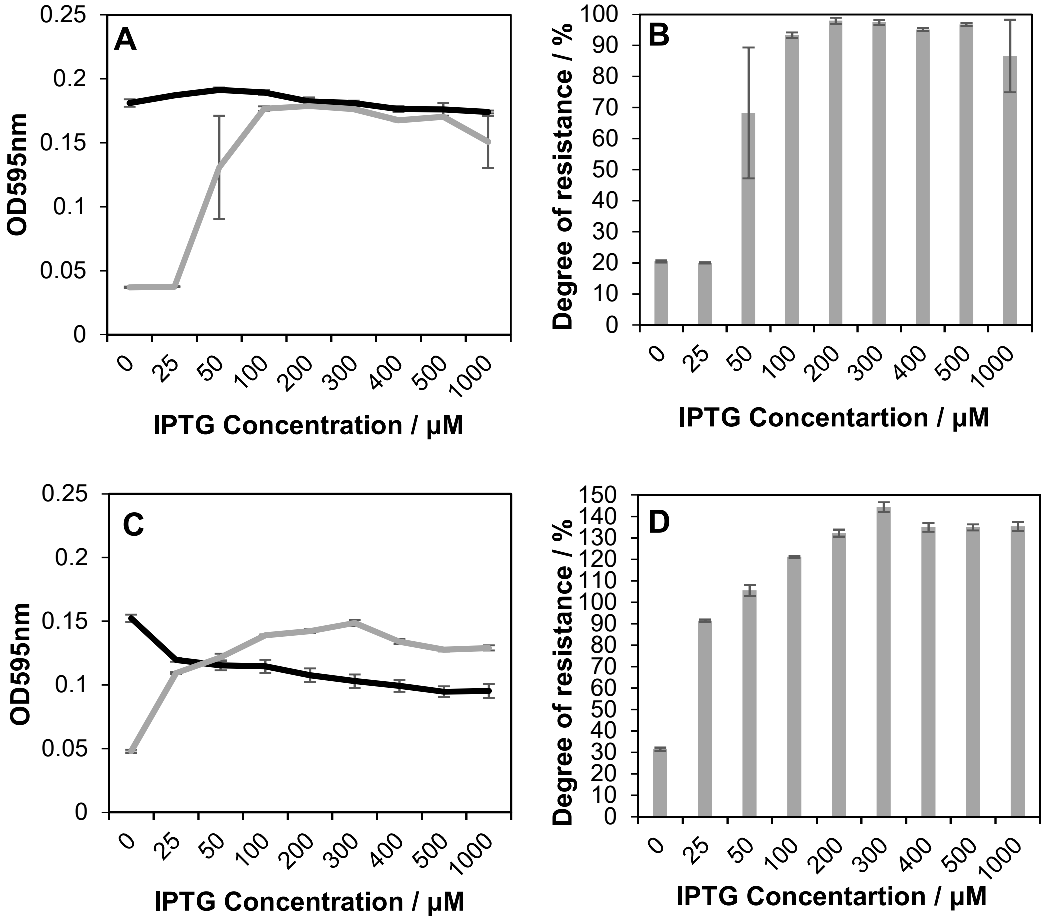

Figure S2. Acquired resistance assay induced by overexpression of target proteins. A: Cell growth of recombinant *E. coli* harboring the *fabI* gene with increasing IPTG concentration. Black line: no triclosan ($OD_{Tc(-)}$). Gray line: 250 nM triclosan ($OD_{Tc(+)}$). B: The degree of resistance, defined as $OD_{Tc(+)}/OD_{Tc(-)}$, against triclosan with FabI overexpression. C: Cell growth of *E. coli* harboring the *folA* gene. Black line: no trimethoprim. Gray line: 125 μM trimethoprim. D: The degree of resistance against trimethoprim.

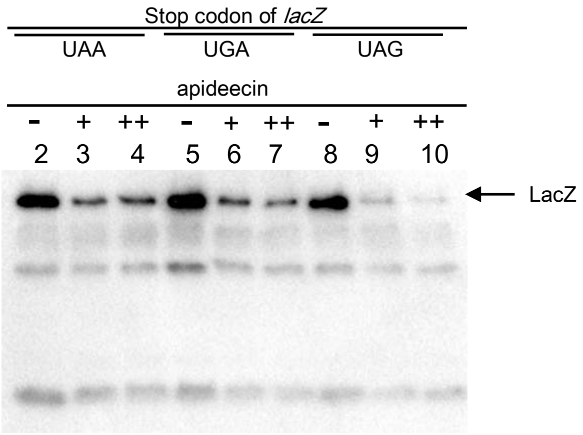

Figure S3. Reproduction of immunoblot result in Figure 4. Experimental conditions are identical to those used in Figure 4. Lanes 2, 3 and 4: MG1655(*lacZ*-UAA), 5, 6 and 7: MG1655(*lacZ*-UGA), and 8, 9 and 10: MG1655(*lacZ*-UAG). Lanes 3, 6, and 9: 250 μM apidaecin was added. Lanes 4, 7 and 10: 500 μM apidaecin was added. The amount of protein was normalized to the OD of the cells.

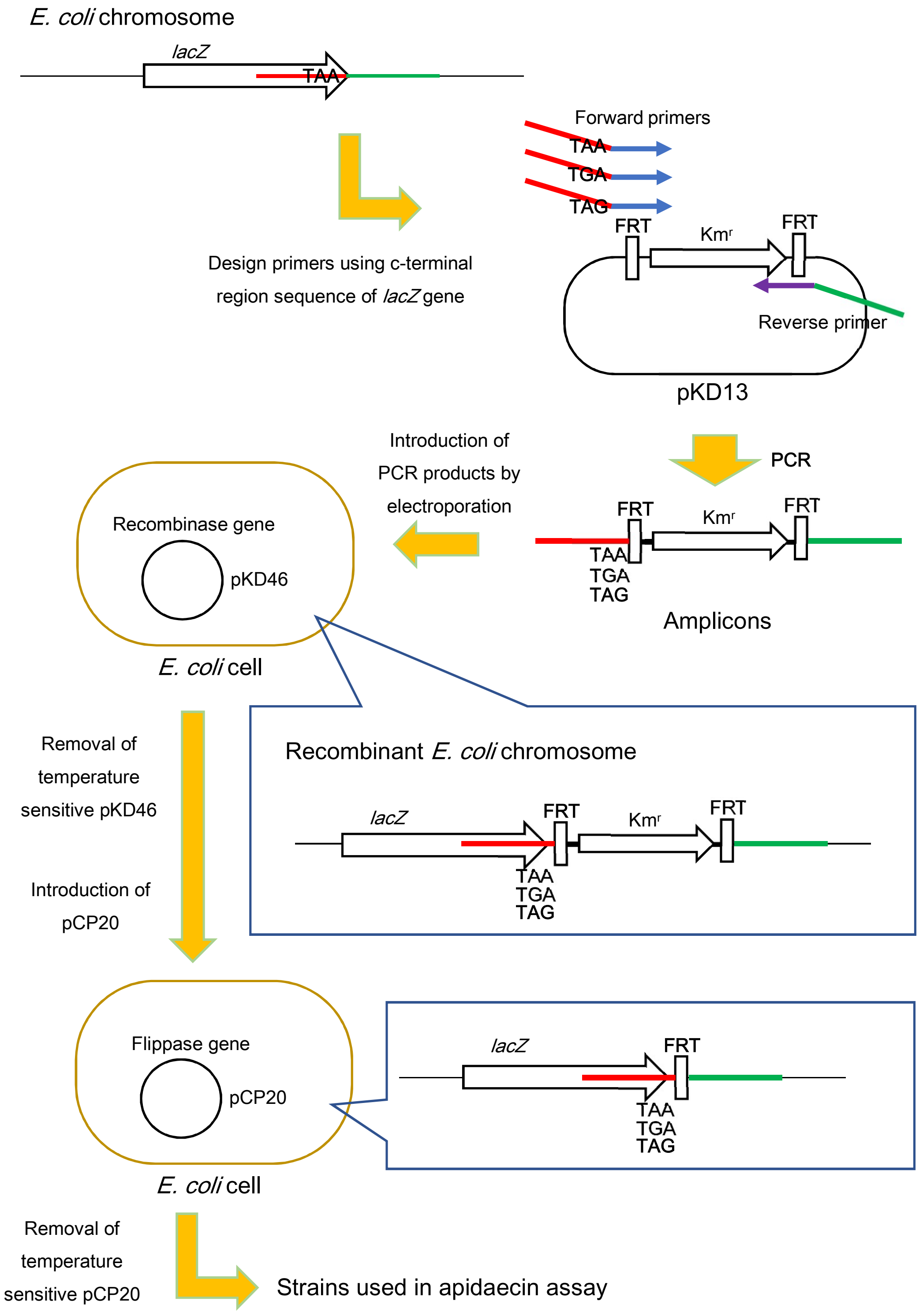

Figure S4. Construction diagram of recombinant *E. coli* possessing altered *lacZ* stop codons. FRT: Flippase recognition target.

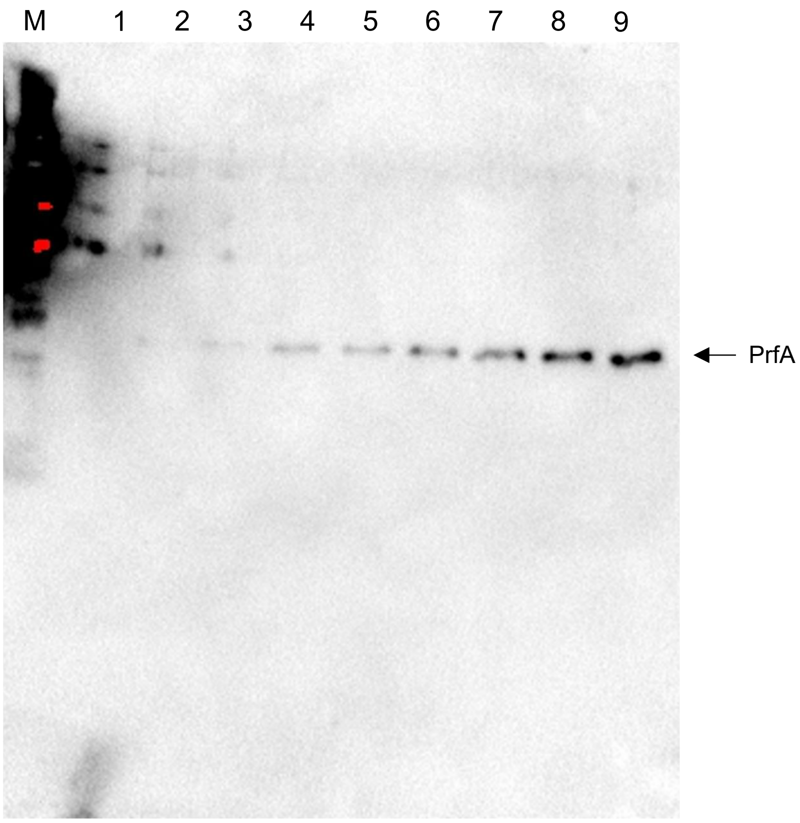

Figure S5. Whole membrane image of immunoblot analysis using anti-His-tag antibody in Figure 3C. Lanes No. 1 to 9 corresponded to IPTG concentration of 0 to 1000 in Figure 3b, respectively. M: size marker. Some of the His-tagged standard proteins acted as a positive control of immunodetection. Red color indicates overexposure that did not influence the detection of His-tagged PrfA.
